# Association Between Sport Participation, Body Composition, Physical Fitness, and Social Correlates Among Adolescents: The PAHL Study

**DOI:** 10.3390/ijerph15122793

**Published:** 2018-12-09

**Authors:** Korcz Agata, Makama Andries Monyeki

**Affiliations:** 1 Department of Didactics of Physical Activity, Faculty of Physical Education, Sport and Rehabilitation, Poznań University of Physical Education, Królowej Jadwigi 27/39, 61-871 Poznań, Poland; 2 Physical Activity, Sport and Recreation Focus Area; Faculty of Health Sciences, North-West University, Potchefstroom 2520, South Africa; andries.monyeki@nwu.ac.za

**Keywords:** physical activity, sport, body composition, physical fitness, social support, adolescents

## Abstract

*Background*: Evidence suggests that social support impacts on participation in sport or physical activity (PA), and is associated with health benefits, although the link is complex and not well understood. The study aim was to examine whether participation in organized sports is related to body composition, physical fitness, and social correlates for PA. *Methods*: Cross-sectional data on 238 adolescents (90 boys and 148 girls), mean age 14.9 ± 0.8 years, who were participants in the Physical Activity and Health Longitudinal Study, were collected. The participants were divided into two groups: sport participation (SP) and non-sport participation (NSP). Height, weight, and triceps and subscapular skinfolds were assessed according to standard procedures. Weight (kg) and height (m^2^) were used to calculate body mass index (BMI), and skinfolds were used to calculate body fat percentage. The European Test of Physical Fitness (EUROFIT) battery of tests was used to assess physical fitness. The standardized International Physical Activity Questionnaire Short Form and Social Support for PA questionnaires were used to obtain information on PA and social correlates for PA, respectively. Participants were asked to choose between participation and non-participation in sport. *Results*: The SP group had lower BMI component values (*p* = 0.011, d = 0.52 for percentage body fat (%BF); *p* = 0.011, d = 0.53 for sum of skinfolds (∑SKF) obtained higher physical fitness scores in selected items (*p* = 0.003, d = 0.64 for sit ups (SUP); *p* < 0.000, d = 0.96 for maximal oxygen consumption VO_2_max) and received higher social support (*p* < 0.001, d = 0.86 for social support (SS)), than the NSP group. The social support received by those participating in sport correlated positively with most fitness components (*p* = 0.013, r^2^ = 18% for bent arm hang (BAH); *p* = 0.000, r^2^ = 12% for sit ups (SUP); *p* = 0.000, r^2^ = 17% for VO_2_max). Physical fitness components were negatively associated with most body composition components for both groups. *Conclusions*: The results provide a better understanding of sport participation in organized sports-related, body composition-related and physical fitness-related associations with changes in social support received by adolescents and may contribute to the development of more accurate promotive strategies to increase children’s and adolescents’ engagement in sport and PA.

## 1. Introduction

Children who are active through sport are more likely to be physically active in adulthood than those who do not participate in childhood sport [1]. Although participation in sport is popular among children, there is evidence indicating a decrease in participation, especially during adolescence. Furthermore, this occurs more among girls than boys [2]. There are two major health-related rationales for focusing attention on adolescent and youth physical activity (PA) [3]: the first is to promote physical health and well-being during these stages of life; the second is to promote PA to enhance future health by increasing the probability of remaining active throughout the lifespan. Worthy of attention is the study of Basterfield et al. [4], which provided evidence that sports club participation is a source of increased PA and decreased levels of adiposity, rather than a consequence of these variables. Therefore, promotion of sport participation needs to focus more on its continuity and prevention of dropping out, than long-term health protective effects.

Concerns exist about children and adolescents who do not meet the PA guideline of 60 min (1 h) or more of PA daily [5,6]. One reason for not meeting this guideline may be that leisure-time PA has changed over the past decades [7]. For instance, sedentary behaviour (SB, such as screen time and non-active transport) has increased drastically, which might partly explain the decrease in PA. PA declines by an estimated 7% per year through adolescence [6] and SB increases with age [8,9]. Hallal et al. [10] estimated that more than 80% of adolescents aged 13–15 years old worldwide fail to meet PA guidelines and found that boys were more active than girls. The general findings summarized in the Global Matrix 2.0: Report Card Grades on the Physical Activity of Children and Youth [11] suggest that “average grades” for overall PA and SB around the world are low, reinforcing the global concern about childhood PA levels. According to Healthy Active Kids South Africa (HAKSA) Report Card [12] on the current status of PA and nutrition in South African youth, more than 50% of children are meeting PA recommendations but children up until the age of 17 are exceeding sedentary recommendations, and sedentary time increases with age. In summary, indisputable evidence of the need to promote PA (i.e., through participation in organized sport) among children and adolescents exists.

It is important to underline the fact that when children and adolescents participate in the recommended level of PA, multiple health benefits accrue [13]. The World Health Organization has advocated that organized sports have an important role to play in reducing the worldwide burden of obesity in childhood and adolescence [14]. The International Olympic Committee has indicated the important role of sport to encourage youth towards behaviour change in order to influence their own health [15]. Existing research evidence shows positive associations between youth sport participation and fulfilling the recommended PA guidelines [16,17] and even with decreased level of adiposity [4]. Sport contributes 55–65% of daily moderate-to-vigorous energy expenditure in youth, who engage in less SB when they participate in sport than those who do not participate [18]. In contrast, Maraques et al. [17] found that participation in sport was unrelated to time spent sedentary. However, community sport participation during leisure time promotes PA for children and adolescents; hence findings associating it with improved physical health, and positive psychological and social health outcomes [19].

According to Sheridan et al. [20], coaches and parents, as well as peers, play a significant role in shaping youth sport experiences both from a positive and negative perspective [20]. Therefore, social factors linked to health benefits must be clearly identified [21], to elucidate their plausible links [22,23,24,25]. Sallis et al. [26] found three variables strongly and consistently associated with a child’s PA index to be: use of afternoon time for sports and PA, enjoyment of physical education (PE) and family support for PA. Social support describes resources provided by interactions with significant others that can influence behaviour [27]. Social support for PA has been identified as an important social correlate of PA in children and adolescents, and even considered by some a key process promoting and facilitating PA among adolescents [28]. Few studies have examined the impact of different types of support (i.e., classmate support, teacher support) on youth PA and have found initial support for this influence [29,30,31]. For instance, results from a study by Duncan et al. [29] revealed that having friends who support and watch youth engage in sports activities is significantly and positively related to youth PA. However, results from one meta-analysis suggest that social support is not a strong predictor of PA in adolescent girls, although parents and friends may have a role in enhancing PA [32]. In a South African study, Cozett et al. [33] found parental influence the strongest predictor of PA overall (stronger than PA self-efficacy, peer influence or perceived PA competence), suggesting that adolescents are more likely to participate in PA if they receive support from their parents. Notably, in a study conducted by Skaal et al. [34], boys had higher social correlates to participate in PA than girls.

Our previous publications on the sample under study in the Physical Activity Health Longitudinal Study (PAHLS) reported findings on body composition, physical fitness, lipid profiles, blood pressure, physical activity and the social correlates of physical activity [34,35,36,37,38], but none of these studies have investigated participation in organized sports (i.e., as a component of PA and not competitive sport) in relation to body composition, physical fitness and PA social correlates. In general, when studying youth PA, the influence of different factors must be considered (i.e., age, gender, type of support, weight status, type of sport and socio-economic status). In addition, family influences, such as parents’ weight status and PA level, have been shown to play a significant role in participation in PA and sport [39]. Therefore, the purpose of this study was to examine whether participation in organized sports is related to body composition, physical fitness and social correlates for PA. We expected that adolescents engaged in sport would have lower values of body mass index (BMI) components (percentage of body fat and skinfold measurements), would be fitter, and would receive more social support (from parents or peers) than non-sport participating adolescents. We assumed that our results would extend the knowledge about existing associations and their directions, especially from developing countries such as South Africa. Furthermore, a better understanding of these associations may contribute to the development of more accurate promotion strategies to increase the engagement of children and adolescents in PA.

## 2. Methods

### 2.1. Study Design and Participants

The article presents findings from cross-sectional (2011) data from PAHLS. Detailed information about the participants, method of data collection and sample size has been published elsewhere [35,38]. The sample comprised 238 adolescents (90 boys and 148 girls) with an average age of 14.9 ± 0.8 years. Participants were asked whether they participated in any organized physical activities or sport, of whom only participants with complete data sets for all variables of interest were included in the analyses. Organized sport was defined as sport activities guided by a coach or teacher (in general by an adult). Responses were dichotomous (yes or no). Based on participants responses about sport participation, for the purpose of this study, the participants were divided into two groups: a sport participation (SP) group (208 participants: 83 boys and 125 girls) and a non-sport participation (NSP) group (30 participants: 7 boys and 23 girls). Gender differences were also investigated.

### 2.2. Anthropometric Measures

Anthropometric measurements of height (cm), weight (kg) and skinfolds (mm) were taken according to the standard procedures described by the International Society for the Advancement Kinanthropometry (ISAK) [40]. BMI, as a measure of body composition, was calculated as body mass/stature^2^ (kg/m^2^). Percentage body fat (%BF) was derived from the sum of triceps and subscapular skinfold (∑SKF) measurements according to an equation developed by Slaughter et al. [41], suitable for use in children from different settings.

### 2.3. Health-Related Physical Fitness Measures

The health-related physical fitness status of participants was determined by assessing their cardiorespiratory endurance, muscle strength and endurance, and flexibility, using the European Test of Physical Fitness [42]. Standing broad jump (SBJ) is designed to measure explosive strength and the results are expressed in centimetres. Bent arm hang (BAH) is designed to measure static arm strength and is expressed in seconds. Sit ups (SUP), designed to measure functional abdominal strength, is expressed as the number of sit ups in 30 s. Cardiorespiratory endurance for maximal oxygen consumption (VO_2_max) was assessed with the 20-m shuttle run test and the result was the level (minute) in which the participant stopped running. Sit and reach (SAR) is designed to measure hamstring flexibility, expressed in centimetres.

### 2.4. Physical Activity and Social Support for Physical Activity

The standardized International Physical Activity Questionnaire Short Form version (IPAQ-SF) [43], and Social Support for Physical Activity questionnaire (SSPA) [44] were used to obtain information on PA and social correlates of PA. The IPAQ-S questionnaire is a valid and reliable tool for assessing PA [45]. Internal consistency was established with the Cronbach’s alpha (α) test. For the IPAQ-SF questionnaire, α = 0.56 and for SSPA questionnaire, α = 0.73. The IPAQ-SF questionnaire comprised of seven questions asking participants about the frequency and time spent sitting, walking and in moderate-to-vigorous physical activity (MVPA) in the last 7 days.

In turn, the SSPA scale included nine questions, asking youth about the different types of support they receive and also give to others during a typical week. Responses were on a three-point scale ranging from 1 (never), 2 (sometimes) to 3 (every day). The questionnaire asked the following questions, “during a typical week”: (1) “How often do you encourage your friends to do physical activity or play sports?”; (2) “How often do your friends encourage you to do physical activity or sports?”; (3) “How often do your friends do physical activities or play sport with you? (4) “How often do your friends tell you that you are doing a good job at a physical activity?”; (5) “Has someone encouraged you to do physical activities or sports?”; (6) “Has someone done a physical activity or played sports with you?”; (7) “Has someone provided transportation to a place where you can do physical activities or play sports?”; (8) “Has someone watched you participate in physical activities or sports?”; and (9) “Has someone told you that you are doing well in physical activity?”. The participants were requested to choose their answer by making a mark or cross next to either never, sometimes or every day. For the purpose of this article we created a new variable named social support (SS), by adding the point scores (1, 2 or 3 points for answers never, sometimes or every day, respectively) Cronbach’s α analyses for internal consistency gave an α score of 0.74.

### 2.5. Ethics

Before data collection for the PAHLS, permission was granted by the District Manager of the Department of Basic Education in Potchefstroom, North West Province, South Africa. The research protocol was approved by the Ethics Committee of North-West University (Ethics number: NWU-0058-01-A1) of the Potchefstroom campus. Parents or guardians gave permission for their children to participate in the study, and participants provided informed assent.

### 2.6. Statistical Analyses

Descriptive statistics are presented as means, standard deviations and frequencies. Overall analyses were performed for sport and non-sport participation, and were also run separately for gender. The *t*-test was used for parametric data and the Mann–Whitney U-test for non-parametric data. The effect size (ES) Cohen’s d (standardized mean differences) was calculated. Guideline values of Cohen’s d = 0.2, 0.5 and 0.8 were regarded as small, medium and large effects, respectively [46] for parametric data and different for non-parametric data (0.1, 0.3 and 0.5). The Spearman’s correlation coefficient was used to measure the strength of association between BMI, %BF, sum of skinfolds (∑SKF) SBJ, BAH, SUP, VO_2_max, SAR, and SS. The correlation effect size (r) was also calculated. Guideline values of correlation effect size ρ = 0.1, 0.3 and 0.5 were regarded as small, medium and large effects, respectively [47]. A simple linear regression was performed separately for two groups (SP group and NSP group) to examine the linear relationship between dependent and predictor variables. The Crosstab procedure was used to calculate the prevalence of social correlates of PA between the SP group and the NSP group by gender, with Pearson Chi-squared used to determine the levels of significant difference. Data analysis was performed using SPSS version 25. P-values are reported for completeness but not interpreted, since a convenience sample, instead of a random sample, was used.

## 3. Results

The descriptive statistics of participants’ characteristics are presented in Table 1, both for the total group and for boys and girls. The mean value for BMI was 20.69 ± 3.87 and 21.59 ± 4.59; %BF 20.00 ± 9.67 and 25.63 ± 11.76; and ∑SKF 25.00 ± 12.40 and 33.06 ± 17.74 for the SP group and NSP group, respectively. For the physical fitness measures, the mean value for SBJ was 165.25 ± 28.84 and 146.23 ± 20.79; BAH 10.18 ± 12.00 and 7.54 ± 10.88; SUP 29.19 ± 10.51 and 22.57 ± 10.08; VO_2_max 34.25 ± 8.11 and 27.29 ± 6.42; and SAR 46.03 ± 8.62 and 45.94 ± 6.91, for the SP group and NSP group, respectively.

In the SP group, girls had significantly (*p* < 0.001) and practically significant (ES ≥ 0.8) higher mean values for %BF and ∑SKF compared with boys. In contrast, boys had significantly (*p* < 0.001) and practically significant (ES ≥ 0.8) higher mean values for the majority of physical fitness items in comparison with girls. Only for SAR did girls obtain significantly (*p* < 0.001) and practically significant (ES ≥ 0.8) higher scores than boys. Among adolescents from the NSP group, practically significant differences were observed for the different gender groups for most physical fitness measures, except for SAR. Boys received significantly (*p* < 0.001) and practically significant (ES ≥ 0.8) higher results than girls for SBJ, BAH, SUP and VO_2_max (*p* < 0.002), while girls showed significantly (*p* < 0.001) and practically significant (ES ≥ 0.8) higher mean values for %BF and ∑SKF. Cohen’s effect size (d) values indicated large and practically significant differences for these measures.

Table 2 presents the body composition and physical fitness profile differences between the adolescents from SP group and NSP group, separately by gender. In general, SP group obtained significantly and practically significant (ES ≥ 0.5) higher scores than NSP group in SBJ (*p* < 0.001), SUP (*p* < 0.01) and VO_2_max (*p* < 0.001). Furthermore, adolescents from SP group scored significantly and practically significantly (ES ≥ 0.5) higher for SS than their peers from NSP group (*p* < 0.001). In turn, adolescents from NSP group had significantly (*p* < 0.05) and practically significant (ES ≥ 0.5) higher body fat (%BF and ∑SKF) than those from SP group. The effect size values indicated large and practically significant differences for these measures.

In boys, we found a statistically significant difference only for SUP (*p* < 0.02), where SP boys scored higher that peers who did not participate in sport.

When comparing girls from the SP and NSP groups, girls who participated in sport obtained significantly and practically significantly (ES ≥ 0.5) higher results in the physical fitness items of SBJ (*p* < 0.01) and VO_2_max (*p* < 0.001), and also scored significantly higher for SS than girls from the NSP group (*p* < 0.01). The effect size values indicated large and practically significant differences for these measures.

Table 3 shows the Spearman’s correlation coefficient between adolescents’ body composition (BMI, %BF and ∑SKF), physical fitness measures (SBJ, BAH, SUP, VO_2_max, and SAR) and social support (SS), separately for SP group and NSP group. In the SP group, BMI was negatively correlated with SBJ (r = −0.23; *p* = 0.01), BAH (r = −0.28; *p* = 0.01) and VO_2_max (r = −0.25; *p* = 0.01). %BF was negatively correlated with SBJ (r = −0.62; *p* = 0.01), BAH (r = −0.66; *p* = 0.01), SUP (r = −0.37; *p* = 0.01) and VO_2_max, (r = −0.66; *p* = 0.01). Furthermore, BMI was positively correlated with SAR (r = 0.19; *p* = 0.01). ∑SKF was negatively correlated with SBJ (r = −0.57; *p* = 0.01), BAH (r = −0.62; *p* = 0.01), SUP (r = −0.31; *p* = 0.01) and VO_2_max (r = −0.59; *p* = 0.01). No significant correlations were found between SS and body composition measures. SS was positively associated with BAH (r = 0.23; *p* = 0.01), SUP (r = 0.34; *p* = 0.01) and VO_2_max (r = 0.40; *p* = 0.01) and negatively with SAR (r = −0.015; *p* = 0.05). The correlation effect size (r) values ranged from small to large significant differences for these measures.

Among adolescents from NSP group, negative correlations were found between BMI and BAH (r = −0.51; *p* = 0.05) and VO_2_max (r = −0.56; *p* = 0.05); and %BF and SBJ (r = −0.49; *p* = 0.05), BAH (r = −0.60; *p* = 0.01) and VO_2_max (r = −0.72; *p* = 0.01). Additionally, ∑SKF was negatively correlated with SBJ (r = −0.41; *p* = 0.01), BAH (r = −0.54; *p* = 0.05) and VO_2_max (r = −68; *p* = 0.01). In this group we reported positive correlation between SS and BMI (r = 0.40; *p* = 0.05) and negative correlation between SS and VO_2_max (r = −0.47; *p* = 0.05). The correlation effect size (r) values ranged from medium to large significant differences for these measures.

Simple linear regression (Table 4) showed that in the SP group SBJ was negatively associated with BMI (β = −0.28, *p* = 0.000, r^2^ = 8%), %BF (β = −0.60, *p* = 0.000, r^2^ = 36%) and ∑SKF (β = −0.52, *p* = 0.00, r^2^ = 28%) and positively associated with ∑SKF (β = 0.14, *p* = 0.047, r^2^ = 2%). BAH was negatively associated with BMI (β = −0.21, *p* = 0.002, r^2^ = 4%), %BF (β = −0.55, *p* = 0.000, r^2^ = 30%) and ∑SKF (β = −0.45, *p* = 0.00, r^2^ = 21%) and positively associated with SS (β = 0.18, *p* = 0.013, r^2^ = 18%). SUP was negatively associated with %BF (β = −0.34, *p* = 0.000, r^2^ = 12%) and ∑SKF (β = −0.28, *p* = 0.000, r^2^ = 8%) and positively associated with SS (β = 0.34, *p* = 0.000, r^2^ = 12%). Also, VO_2_max was inversely associated with BMI (β = −0.30, *p* = 0.000, r^2^ = 9%), %BF (β = −0.67, *p* = 0.000. r^2^ = 45%) and ∑SKF (β = −0.56, *p* = 0.000, r^2^ = 56%) and positively associated with SS (β = 0.41, *p* = 0.000, r^2^ = 17%). SAR was positively associated with %BF (β = 0.17, *p* = 0.015, r^2^ = 3%).

In the NSP group, we found that SBJ was negatively associated with %BF (β = −0.45, *p* = 0.012, r^2^ = 21%) and ∑SKF (β = −0.39, *p* = 0.035, r^2^ = 15%). BAH was negatively associated with BMI (β = −0.41, *p* = 0.024, r^2^ = 17%), %BF (β = −0.47, *p* = 0.009, r^2^ = 22%) and SKK (β = −0.40, *p* = 0.030, r^2^ = 16%). VO_2_max was inversely associated with BMI (β = −0.41, *p* = 0.040, r^2^ = 17%), %BF (β = −0.68, *p* = 0.000, r^2^ = 46%) and ∑SKF (β = −0.58, *p* = 0.002, r^2^ = 58%). SAR was negatively associated with BMI (β = −0.49, *p* = 0.009, r^2^ = 24%) and ∑SKF (β = −0.40, *p* = 0.036, r^2^ = 16%).

Table 5 presents the results of the social correlates of PA for the SP and NSP groups, separately for boys and girls. The SP group of girls significantly showed more attention when they had been watched by someone; and while they were participating in PA or sport (38.7% every day and 51.6% sometimes) than girls from the NSP group (39.1% never; χ^2^ = 0.001).

In the group of boys, we found more significant differences between those who participated and those who do not participate in sport than in girls. The SP and NSP groups of boys indicated similar percentages of friends who were participating in PA or playing sport with them every day (54.3% and 57.1%, respectively); however, significant differences were found in the answers “sometimes” and “never” (Crosstabs analysis, χ^2^ = 0.016), where 50.6% of SP boys selected sometimes and 57.1% of NSP boys selected never. The SP group of boys indicated significantly often (42.0% every day and 50.6% sometimes) that their friends tell them that they are doing a good job at PA, more than boys from the NSP group (57.1% never) (Crosstabs analysis, χ^2^ = 0.000).

The SP and NSP groups of boys demonstrated significant differences in terms of support from someone who provided transportation to a place where they could participate in PA or plays sports (Crosstabs analysis, χ^2^ = 0.040), with 27.2% of boys from the SP group indicating that they had transport ‘every day’, 45.7% of them selecting “sometimes’ and 71.4% of boys selecting the answer “never’. None of the NSP boys indicated that someone provided transportation “every day”.

Finally, the SP group of boys significantly indicated that someone told them that they are doing well in PA “often” (44.4% every day and 49.4% sometimes), compared with boys from the NSP group (57.1% never) (Crosstabs analysis, χ^2^ = 0.000).

## 4. Discussion

This study examined whether participation in organized sports is related to body composition, physical fitness and social correlates of PA. It was hypothesized that there would be a positive association between physical fitness and social support for PA among adolescents from a sport-participation group, that would contribute to a decrease in body composition components.

The results show significantly high and positive correlations for body composition components (BMI and %BF; ∑SKF and BMI; %BF and ∑SKF), both in SP and NSP groups. However, this finding is not surprising, as previous studies have shown that all stated variables are indicators of fatness [37,48]. Additionally, significant negative associations between selected physical fitness items (SBJ, BAH, VO_2_max) and body composition components (BMI, %BF, ∑SKF), both for the SP group and NSP group, were found (except for the association between SBJ and BMI in the NSP group). However, those associations were in general stronger in the SP group. In the same group of adolescents, we also reported significantly more negative associations between physical fitness and body composition (between SUP and %BF, SUP and ∑SKF, and SAR and %BF) compared with the NSP group. These results highlight the important contribution of sport participation to numerous health benefits, such as improved aerobic fitness, muscular strength and endurance, increased bone mineral density and a more favourable cardiovascular risk factor profile [13,49].

The results also show significant positive associations between selected physical fitness items (BAH, SUP, VO_2_max) and SS, but only for adolescents from the SP group. Similar findings to those of our study have been shown in other studies, where, among other factors, sport participation may influence cardiorespiratory fitness, which appears to have an impact on BMI and other metabolic factors [50]. On the contrary, Marques et al. [49] indicated that participation in sports was unrelated to BMI. However, it may be related to the form of the PA (sport discipline), such as the demands of specific sports, and to differences between individual players and coaching styles [51].

We found other significant group and group-gender differences in our study. We observed differences in physical fitness between males and females, both in the SP group and the NSP group. Boys from both groups scored higher in the majority of the physical fitness items, except SAR, where girls, but only from the SP group, obtained higher scores. Our results are in line with previous research that has consistently shown that boys score higher in physical fitness than girls [35,52] and that girls in particular are more flexible at all ages than boys [35]. Furthermore, in our study, the gender difference was also observed in %BF, with girls having a higher %BF than boys. Other researchers have also proved that boys have greater muscle mass and less fat than girls [53,54].

In our findings, it was apparent that adolescents’ participation in PA was affected by different aspects in the different groups depending on involvement in sport or not. We identified that encouragement (social support) might be a key contributor to adolescents being active or participating in sport. Adolescents from the SP group reported receiving higher support (in a different context) than their peers from the NSP group. Despite far-reaching research examining the relationship between social support and participation in PA, inconsistencies have emerged. For instance, in the study of Higgins et al. [55], girls reported more social support, while in other studies boys were the ones who obtained more support [56,57]. Furthermore, Prochaska et al. [58] found no association between monitored PA and social support. More research is therefore necessary to evaluate the social support given by parents, peers or teachers and its impact on adolescents.

Both the girls and boys who participated in sport reported significantly higher social support than their counterparts in the NSP groups (19.43 ± 3.11 vs. 17.10 ± 3.31; 20.71 ± 3.09 vs.16.86 ± 4.67, respectively, girls and boys). Overall, social support was a strong predictor of physical fitness (except for SAR) only in the SP group. This finding confirms that social support is connected with physical fitness and sport participation. Girls from the SP group showed more attention while participating in PA/sport than girls from the other group. However, boys from both the SP and the NSP groups showed significant differences with regard to “support from someone who provided transportation to a place where they could do PA or plays sports”, to the disadvantage of those boys who do not participate in sport. Furthermore, we found that boys who participate in sport often got feedback from their friends that they are doing well at PA. Boys from the SP group were significantly more likely to have friends participating in PA or playing sport. Similar results were found in a study by Duncan et al. [29], which revealed that having friends who support and watch youth engage in sports activities is significantly and positively related to youth PA. In general, a growing body of research indicates that social support exerts a strong influence on adolescent MVPA [59] and is an important contributor to increased PA among adolescents [60,61]. In a study by Kubayi et al. [62], sport participation among learners of the secondary schools in the rural areas of Limpopo Province in South Africa was found likely to increase when they received informational, tangible, emotional support from their parents and peers.

Limitations need to be taken into consideration when interpreting these results. First, participation in organized sport was self-reported, which may have influenced the direction of the current findings. Second, we did not take into consideration the type of sport performed, frequency or duration. We only considered weekly practice of PA that involved training sessions organized by adults. Third, the SP and NSP group sample sizes were unequal. Interestingly, most of the examined youth participated in sports activities (208 of the 238 youth), which may be related to the PE system in South African schools, where PE is part of a multidimensional learning area known as Life Orientation (with only one hour per week allocated to PE teaching) [63]. We presume that one hour of Life Orientation combined with other unrelated PA components may be insufficient for young people to achieve the daily required PA. Furthermore, contrary to the findings of previous studies [49,64], more girls than boys were involved in organized sport (60% of girls and 40% of boys). Fourth, the design of this study was cross-sectional. Nevertheless, the findings contribute important information about the adolescent population of Tlokwe Local Municipality, North West Province, South Africa. The strength of the study is that, to our knowledge, it is one of few of its kind to assess the relationships between sport participation, body composition, physical fitness and social correlates in South Africa. Future studies need to consider the social context while interpreting research findings on associations between these correlates. A better understanding of the correlates of PA—or sport participation—may help in the promotion of PA among these groups.

## 5. Conclusions

Our findings indicated that participation in organized sport was associated with higher physical fitness, higher social support and lower body composition among adolescents. Sport participants were fitter, had lower body composition and obtained more social support compared with non-sport participants. The participation of adolescents in organized sports may be a way to minimize increases in body composition, and greater social support (from parents and peers) may enable more adolescents to participate in sport. Participation in organized sport conveys lasting benefits that are considered attributes of a healthy lifestyle. Therefore, the youth must be encouraged to participate in sport or PA and parents taught how to support their children. More accurate promotive strategies are needed to increase adolescent engagement in sport and general PA.

## Figures and Tables

**Table 1:** Mean and standard deviation of anthropometric, body composition and physical fitness characteristics of adolescents by sport participation and gender.

	SP Group					NSP Group				
	Total Group	Boys	Girls	*p*-Value	d	Total Group	Boys	Girls	*p*-Value	d
Variables	(*N* = 208)	(*n* = 83)	(*n* = 125)			(*N* = 30)	(*n* = 7)	(*n* = 23)		
	M ± SD	M ± SD	M ± SD			M ± SD	M ± SD	M ± SD		
Age (years)	14.88 ± 0.84	14.92 ± 0.76	14.86 ± 0.90	0.599	0.07	14.80 ± 0.76	14.73 ± 0.98	14.83 ± 0.70	0.864	0.12
Stature (cm)	161.57 ± 9.13	166.78 ± 9.32	158.10 ± 7.17	<0.001	1.05	157.19 ± 6.41	159.51 ± 7.60	156.48 ± 6.01	0.249	0.44
Body mass (kg)	54.32 ± 12.60	56.39 ± 12.58	52.95 ± 12.47	0.054	0.27	53.59 ± 12.90	49.40 ± 9.28	54.86 ± 13.73	0.418	0.47
BMI (kg/m^2^)	20.69 ± 3.87	20.10 ± 3.26	21.08 ± 4.21	0.060	0.26	21.59 ± 4.59	19.27 ± 2.32	22.29 ± 4.91	0.091	0.83
%BF	20.00 ± 9.67	12.84 ± 7.88	24.76 ± 7.60	<0.001	1.54	25.63 ± 11.76	13.29 ± 4.74	29.39 ± 10.62	<0.001	2.10
∑SKF (mm)	25.00 ± 12.40	18.37 ± 8.71	29.41 ± 12.56	<0.001	1.04	33.06 ± 17.74	18.76 ± 5.02	37.41 ± 17.98	0.004	1.62
SBJ (cm)	165.25 ± 28.84	187.12 ± 25.10	150.61 ± 20.84	<0.001	1.59	146.23 ± 20.79	170.14 ± 20.54	138.96 ± 14.75	0.001	1.77
BAH (s)	10.18 ± 12.00	19.05 ± 14.01	4.36 ± 4.99	<0.001	1.55	7.54 ± 10.88	18.70 ± 9.16	4.15 ± 9.03	0.001	1.60
SUP (s)	29.19 ± 10.51	36.10 ± 7.29	24.53 ± 9.78	<0.001	1.35	22.57 ± 10.08	29.14 ± 5.90	20.57 ± 10.32	0.069	1.06
Predicted VO_2_max	34.25 ± 8.11	41.17 ± 6.27	29.22 ± 4.99	<0.001	2.12	27.29 ± 6.42	37.12 ± 7.19	24.83 ± 3.05	0.006	2.40
SAR (cm)	46.03 ± 8.62	41.70 ± 9.30	48.96 ± 6.73	<0.001	0.90	45.94 ± 6.91	43.92 ± 8.35	46.50 ± 6.58	0.218	0.34

SP = sport participation group; NSP = non-sport participation group; M = mean; SD = standard deviation; *d* = sample effect size (Cohen); BMI = body mass index; %BF = percentage body fat; ∑SKF = sum of skinfolds; SBJ = standing broad jump; BAH = bent arm hang; SUP = sit ups; SAR = sit and reach, VO_2_max = maximal oxygen consumption.

**Table 2:** Mean and standard deviation of body composition and physical fitness differences between adolescents from sport participation and non-sport participation groups by gender.

	Total				Boys				Girls			
Variables	SP Group	NSP Group	*p*-Value	d	SP Group	NSP Group	*p*-Value	d	SP Group	NSP Group	*p*-Value	d
Body mass (kg)	54.32 ± 12.60	53.59 ± 12.90	0.816	0.06	56.39 ± 12.58	49.40 ± 9.28	0.195	0.64	52.95 ± 12.47	54.86 ± 13.73	0.483	0.14
BMI (kg/m^2^)	20.69 ± 3.88	21.59 ± 4.59	0.358	0.21	20.10 ± 3.26	19.27 ± 2.32	0.474	0.30	21.08 ± 4.21	22.29 ± 4.91	0.256	0.26
%BF	20.00 ± 9.67	25.63 ± 11.76	0.011	0.52	12.84 ± 7.88	13.29 ± 4.74	0.386	0.07	24.76 ± 7.60	29.39 ± 10.62	0.031	0.51
∑SKF (mm)	25.00 ± 12.40	33.06 ± 17.74	0.011	0.53	18.37 ± 8.71	18.76 ± 5.02	0.320	0.06	29.41 ± 12.56	37.41 ± 17.62	0.031	0.53
SBJ (cm)	165.25 ± 28.84	146.23 ± 20.79	<0.001	0.77	187.12 ± 25.10	170.14 ± 20.54	0.067	0.74	150.61 ± 20.84	138.96 ± 14.75	0.006	0.65
BAH (s)	10.18 ± 12.00	7.54 ± 10.88	0.099	0.23	19.05 ± 14.01	18.70 ± 9.16	0.732	0.03	4.36 ± 4.99	4.15 ± 9.03	0.198	0.03
SUP (s)	29.19 ± 10.51	22.57 ± 10.08	0.003	0.64	36.10 ± 7.29	29.14 ± 5.90	0.015	1.06	24.53 ± 9.78	20.57 ± 10.32	0.185	0.40
Predicted VO_2_max	34.25 ± 8.11	27.29 ± 6.42	<0.001	0.96	41.17 ± 6.27	37.12 ± 7.19	0.236	0.60	29.22 ± 4.99	24.83 ± 3.05	<0.001	1.09
SAR (cm)	46.03 ± 8.62	45.94 ± 6.91	0.858	0.01	41.70 ± 9.30	43.92 ± 8.35	0.722	0.25	48.96 ± 6.73	46.50 ± 6.58	0.154	0.37
SS (pts)	19.95 ± 3.16	17.04 ± 3.60	<0.001	0.86	20.71 ± 3.09	16.86 ± 4.67	0.022	0.99	19.43 ± 3.11	17.10 ± 3.31	0.006	0.72

SP = sport participation group; NSP = non-sport participation group; M = mean; SD = standard deviation; d = sample effect size (Cohen); B = boys; G = girls; BMI = body mass index; %BF = percentage body fat; ∑SKF = sum of skinfolds; SBJ = standing broad jump; BAH = bent arm hang; SUP = sit ups; SAR = sit and reach; SS = social support, VO_2_max = maximal oxygen consumption.

**Table 3:** Spearman’s correlation coefficient (*r*) between body composition, physical fitness, and social correlates for sport participation and non-sport participation groups.

**SP Group**									
	**BMI**	**%BF**	**SKK**	**SBJ**	**BAH**	**SUP**	**VO_2_max**	**SAR**	**SS**
BMI (kg/m^2^)									
%BF	0.67 **								
∑SKF (mm)	0.73 **	0.98 **							
SBJ (cm)	−0.23 **	−0.62 **	−0.57 **						
BAH (s)	−0.28 **	−0.66 **	−0.62 **	0.66 **					
SUP (s)	0.03	−0.37 **	−0.31 **	0.53 **	0.60 **				
VO_2_max	−0.25 **	−0.66 **	−0.59 **	0.65 **	0.64 **	0.60 **			
SAR (cm)	0.00	0.19 **	0.13	−0.24 **	−0.25 **	−0.36 **	−0.31 **		
SS (pts)	0.06	−0.05	0.01	0.14	0.23 **	0.34 **	0.40 **	−0.15 *	
	**NSP Group**								
	BMI	%BF	SKK	SBJ	BAH	SUP	VO_2_max	SAR	SS
BMI (kg/m^2^)									
%BF	0.80 **								
∑SKF (mm)	0.83 **	0.99 **							
SBJ (cm)	−0.29	−0.49 **	−0.41 *						
BAH (s)	−0.51 **	−0.60 **	−0.54 **	0.59 **					
SUP (s)	−0.01	−0.23	−0.17	0.64 **	0.38 *				
VO_2_max	−0.56 **	−0.72 **	−0.68 **	0.53 **	0.63 **	0.24			
SAR (cm)	−0.28	−0.14	−0.20	−0.12	0.02	−0.22	0.16		
SS (pts)	0.40 *	0.29	0.31	0.01	−0.19	−0.03	−0.47 *	−0.21	

SP = sport participation group; NSP = non-sport participation group; BMI = body mass index; %BF = percentage body fat; ∑SKF = sum of skinfolds; SBJ = standing broad jump; BAH = bent arm hang; SUP = sit ups; SAR = sit and reach; SS = social support, VO_2_max = maximal oxygen consumption. * Correlation is significant at the 0.05 level (two-tailed). ** Correlation is significant at the 0.01 level (two-tailed).

**Table 4:** Simple linear regression coefficient (β), 95% CI and p-values for associations between physical fitness measures and prediction variables.

							SP Group								
Predictors	SBJ			BAH			SUP			VO_2max_			SAR		
	β	95% CI	*p*-Value	β	95% Cl	*p*-Value	β	95% CI	*p*-Value	β	95% CI	*p*-Value	β	95% CI	*p*-Value
BMI (kg/m^2^)	−0.28	−3.06; −1.10	0.000	−0.21	−1.08; −0.24	0.002	−0.03	−0.45; 0.29	0.669	−0.30	−1.01; −0.34	0.000	0.01	−0.29; 0.32	0.927
%BF	−0.60	−2.12; −1.46	0.000	−0.55	−0.82; −0.54	0.000	−0.34	−0.51; −0.23	0.000	−0.67	−0.68; −0.48	0.000	0.17	0.03; 0.27	0.015
∑SKF (mm)	−0.52	−1.49; −0.95	0.000	−0.45	−0.56; −0.32	0.000	−0.28	−0.35; −0.13	0.000	−0.56	−0.48; −0.30	0.000	0.12	−0.01; 0.18	0.098
SS (pts)	0.14	0.02; 2.56	0.047	0.18	0.14; 1.20	0.013	0.34	−0.70; 1.59	0.000	0.41	0.74; 1.54	0.000	−0.12	−0.72; 0.06	0.100
	**NSP Group**														
BMI (kg/m^2^)	−0.23	−2.76; 0.64	0.214	−0.41	−1.81; −0.13	0.024	−0.14	−1.14; 0.54	0.469	−0.41	−1.07; −0.03	0.040	−0.49	−1.22; −0.20	0.009
%BF	−0.45	−1.41; −0.19	0.012	−0.47	−0.75; −0.12	0.009	−0.32	−0.59; 0.04	0.088	−0.68	−0.52; −0.19	0.000	−0.34	−0.41; 0.02	0.074
∑SKF (mm)	−0.39	−0.87; −0.03	0.035	−0.40	−0.46; −0.02	0.030	−0.32	−0.39; 0.03	0.087	−0.58	−0.32; −0.08	0.002	−0.40	−0.29; −0.01	0.036
SS (pts)	0.12	−1.59; 3.02	0.530	−0.02	−1.31; 1.17	0.914	−0.01	−1.09; 1.06	0.975	0.45	−1.65; 0.21	0.124	0.43	−1.20; 0.60	0.500

SP = sport participation group; NSP = non-sport participation group; BMI = body mass index; %BF = percentage body fat; ∑SKF = sum of skinfolds; SBJ = standing broad jump; BAH = bent arm hang; SUP = sit ups; SAR = sit and reach; SS = social support; VO_2_max = maximal oxygen consumption; CI = confidence interval.

**Table 5:** Percentage (%) social correlates of physical activity by group and gender.

	SP Group					NSP Group			
Social Correlates		Never	Sometimes	Everyday		Never	Sometimes	Everyday	χ^2^
		*N* (%)	*N* (%)	*N* (%)		*N* (%)	*N* (%)	*N* (%)	
During a typical week:									
1. How often do you encourage your friend to do PA or play sports?	*B*	5 (6.1)	48 (58.5)	29 (35.4)	*B*	2 (28.6)	4 (57.1)	1 (14.3)	0.082
	*G*	10 (8.1)	84 (67.7)	30 (24.2)	*G*	2 (8.7)	20 (87.0)	1 (4.3)	0.279
2. How often do your friends encourage you to do PA or sports?	*B*	5 (6.2)	51 (61.4)	25 (30.9)	*B*	2 (28.6)	4 (57.1)	1 (14.3)	0.095
	*G*	30 (24.2)	67 (54.0)	27 (21.8)	*G*	6 (26.1)	16 (69.6)	1 (4.3)	0.136
3. How often do your friends do PA or play sport with you?	*B*	3 (3.7)	34 (42.0)	44 (54.3)	*B*	2 (28.6)	1 (14.3)	4 (57.1)	0.016
	*G*	12 (9.7)	65 (52.4)	47 (37.9)	*G*	5 (22.7)	11 (50.0)	6 (27.3)	0.279
4. How often do your friends tell you that you are doing a good job at PA?	*B*	6 (7.4)	41 (50.6)	34 (42.0)	*B*	4 (57.1)	1 (14.3)	2 (28.6)	0.000
	*G*	26 (21.1)	66 (53.7)	31 (25.2)	*G*	8 (34.8)	11 (47.8)	4 (17.4)	0.252
5. Has someone encouraged you to do PA or sports?	*B*	13 (16.0)	40 (49.4)	28 (34.6)	*B*	-	6 (85.7)	1 (14.3)	0.169
	*G*	13 (10.7)	64 (52.9)	44 (36.4)	*G*	7 (30.4)	10 (43.5)	6 (26.1)	0.084
6. Has someone done a PA or played sports with you?	*B*	5 (6.3)	36 (45.0)	39 (48.8)	B	2 (28.6)	3 (42.9)	2 (28.6)	0.103
	*G*	8 (6.6)	70 (57.4)	44 (36.1)	*G*	4 (19.0)	12 (57.1)	5 (23.8)	0.299
7. Has someone provided transportation to a place where you can do PA or play sports?	*B*	22 (27.2)	37 (45.7)	22(27.2)	*B*	5 (71.4)	2 (28.6)	-	0.040
	*G*	46 (37.1)	57 (46.0)	21 (16.9)	*G*	12(52.2)	5 (21.7)	6 (26.1)	0.061
8. Has someone watched you participate in PA or sports?	*B*	8 (9.9)	39 (48.1)	34 (42.0)	*B*	2 (28.6)	3 (42.9)	2 (28.6)	0.316
	*G*	12 (9.7)	64 (51.6)	48 (38.7)	*G*	9 (39.1)	8 (34.8)	6 (26.1)	0.001
9. Has someone told you that you are doing well in PA?	*B*	5 (6.2)	40 (49.4)	36 (44.4)	*B*	4 (57.1)	1 (14.3)	2 (28.6)	0.000
	*G*	12 (9.8)	63 (51.2)	48 (39.0)	*G*	6 (26.1)	13 (56.5)	4 (17.4)	0.099

SP = sport participation group; NSP = non-sport participation group; *B* = boys; *G* = girls; χ^2^ = Pearson Chi-squared.
